# Large phonon drag thermopower boosted by massive electrons and phonon leaking in LaAlO_3_/LaNiO_3_/LaAlO_3_ heterostructure

**DOI:** 10.1021/acs.nanolett.1c03143

**Published:** 2021-10-28

**Authors:** Masatoshi Kimura, Xinyi He, Takayoshi Katase, Terumasa Tadano, Jan M. Tomczak, Makoto Minohara, Ryotaro Aso, Hideto Yoshida, Keisuke Ide, Shigenori Ueda, Hidenori Hiramatsu, Hiroshi Kumigashira, Hideo Hosono, Toshio Kamiya

**Affiliations:** † Laboratory for Materials and Structures, Institute of Innovative Research, Tokyo Institute of Technology, 4259 Nagatsuta, Midori, Yokohama 226-8503, Japan; ‡ PRESTO, Japan Science and Technology Agency, 7 Gobancho, Chiyoda, Tokyo 102-0076, Japan; § National Institute for Materials Science, Sengen, Tsukuba 305-0047, Japan; ∥ Institute of Solid State Physics, Vienna University of Technology, Wiedner Hauptstrasse 8-10, A-1040 Vienna, Austria; ⊥ Research Institute for Advanced Electronics and Photonics, National Institute of Advanced Industrial Science and Technology, Tsukuba, Ibaraki 305-8568, Japan; # Department of Applied Quantum Physics and Nuclear Engineering, Kyushu University, Fukuoka, Fukuoka 819-0395, Japan; ∇ The Institute of Scientific and Industrial Research, Osaka University, 8-1 Mihogaoka, Ibaraki, Osaka 567-0047, Japan; ○ Research Center for Functional Materials, National Institute for Materials Science, Namiki, Tsukuba 305-0044, Japan; ● Research Center for Advanced Measurement and Characterization, National Institute for Materials Science, Tsukuba 305-0047, Japan; △ Synchrotron X-ray Station at SPring-8, National Institute for Materials Science, 1-1-1 Sayo, Hyogo, 679-5148, Japan; ▲ Materials Research Center for Element Strategy, Tokyo Institute of Technology, 4259 Nagatsuta, Midori, Yokohama 226-8503, Japan; □ Photon Factory, Institute of Materials Structure Science, High Energy Accelerator Research Organization, Tsukuba, Ibaraki 305-0801, Japan; ■ Institute of Multidisciplinary Research for Advanced Materials, Tohoku University, Sendai 980-8577, Japan

**Keywords:** Thermoelectrics, Thin film heterostructure, Strongly correlated electron oxide, Transition-metal oxide

## Abstract

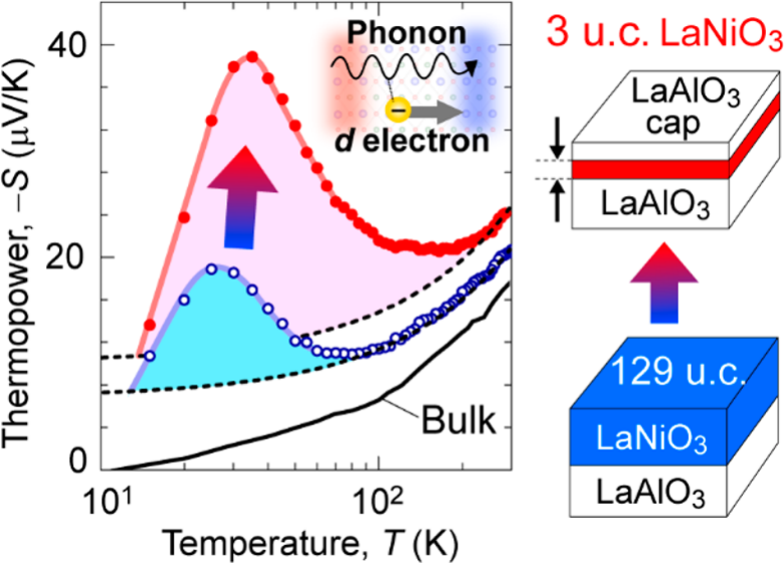

An unusually large thermopower (*S*) enhancement is induced by heterostructuring thin films of the strongly correlated electron oxide LaNiO_3_. The phonon-drag effect, which is not observed in bulk LaNiO_3_, enhances *S* for thin films compressively strained by LaAlO_3_ substrates. By a reduction in the layer thickness down to three unit cells and subsequent LaAlO_3_ surface termination, a 10 times *S* enhancement over the bulk value is observed due to large phonon drag *S* (*S*_g_), and the *S*_g_ contribution to the total *S* occurs over a much wider temperature range up to 220 K. The *S*_g_ enhancement originates from the coupling of lattice vibration to the d electrons with large effective mass in the compressively strained ultrathin LaNiO_3_, and the electron–phonon interaction is largely enhanced by the phonon leakage from the LaAlO_3_ substrate and the capping layer. The transition-metal oxide heterostructures emerge as a new playground to manipulate electronic and phononic properties in the quest for high-performance thermoelectrics.

The atomic-scale control of thin-film heterostructures based on transition-metal oxides (TMOs) is a fruitful way to elicit exotic electronic properties that are not accessible in bulk materials.^1−3^ By control of the layer thickness and chemical composition, band structures and electronic correlations can be largely tailored, which allows a systematic tuning of the electronic properties such as the metal–insulator transitions, magnetic order, and superconductivity. On the other hand, recent studies have emphasized the roles of electron–phonon (e-ph) interactions; i.e., coupling of charge carriers and phonons at the interface of different materials also plays a crucial role, potentially leading to exotic physical properties.^4,5^ However, the effect of e-ph interactions on electronic properties in TMO heterostructures is still not fully explored on the atomic-scale.

Here we report the discovery of an unusually large thermopower enhancement by a phonon drag effect in ultrathin compressively strained LaNiO_3_ (LNO) films, which is further enhanced by phonon leaking with large penetration depth due to a heterostructure composed of similar perovskite-type structure oxides: LaAlO_3_ (LAO) capping layer/LNO ultrathin film/LAO substrate. It is known that the thermopower (*S*) is determined by the electrostatic potential generated by a temperature gradient and observed as the sum of the electron diffusion *S* (*S*_d_) and an additional phonon drag *S* (*S*_g_): i.e., *S* = *S*_d_ + *S*_g_. The *S*_g_ appears due to the momentum exchange between charge carriers and nonequilibrium lattice vibrations and can give rise to a large increase in *S*. Phenomenologically, *S*_g_ is given by , where *μ* is the carrier mobility, *ν*_λ_ is the phonon group velocity, *l*_*λ*_ is the phonon mean free path, and *f* is the fraction of the crystal momentum lost by the lattice vibrations that are transferred to the charge carriers, in the nondegenerated regime.^6^ Therefore, the phonon-drag effect is proportional to the strength of the e-ph coupling and the relaxation time of the phonons coupled to charge carriers.

LNO is a good platform to attempt enhancing *S*_g_ by manipulating the heterostructure. Bulk LNO is a strongly correlated metal with an electron effective mass (m*/*m*_0_) as large as ∼10.^7,8^ It is reported that metallic LNO films compressively strained on LAO substrates show a notable *S*_g_ at low *T* ≈ 25 K,^9,10^ which cannot be seen in bulk LNO.^11^ When the LNO film thickness is reduced down to a few unit cells (u.c.) of the perovskite lattice, the 3d electron interactions of the Ni^3+^ (t_2g_^6^e_g_^1^) ions become predominant and a transition from a metallic to an electron-localized insulating state occurs at a critical thickness.^12−20^ It is also reported that the LNO surface layer has a strong polar distortion coupled with the Ni–O_6_ octahedra rotation. A surface capping by a few u.c. LAO insulating layers recovers a metallic state by suppressing the structural distortion.^15^ The good tunability of electronic correlations in ultrathin LNO films offers the possibility to further enhance the *S*_g_.

In addition, the recent discovery of phonon leaking from a substrate material into a ultrathin conducting layer, e.g., in Bi_2_Te_3_/Al_2_O_3_^21^ and FeSe/SrTiO_3_,^22^ suggests an additional mechanism to further enhance *S*_g_. Thus, we expect a similar enhancement by phonon leaking would be possible by heterostructuring a ultrathin LNO film with an LAO substrate and capping layer because these oxides have similar perovskite-type structures and easily form a high-quality heterostructure.

We herein investigate the effects of layer thickness and surface termination on the *S*_g_ of LNO films that are compressively strained on a LAO substrate (Figure 1a). The 129–3 u.c. thick LNO epitaxial films with step and terrace surfaces were grown at 700 °C on a (001) LAO single crystal by pulsed laser deposition. The LAO-capped LNO film was fabricated by first depositing a 3 u.c. LNO film, followed by growing a 10 u.c. LAO capping layer. The LNO film grows coherently with compressive strain (the pseudocubic out-of-plane (*c*) and in-plane (*a*) lattice parameter ratio is *c*/*a* = 1.026) on the LAO substrate (Figure 1b and Figure S1). In addition, for the LAO-capped 3 u.c. LNO film, the trilayer structure of LAO/LNO/LAO grows coherently with strain (Figure 1c). The chemical composition and electronic state analyses are summarized in Figures S2–S5.

**Figure 1:**
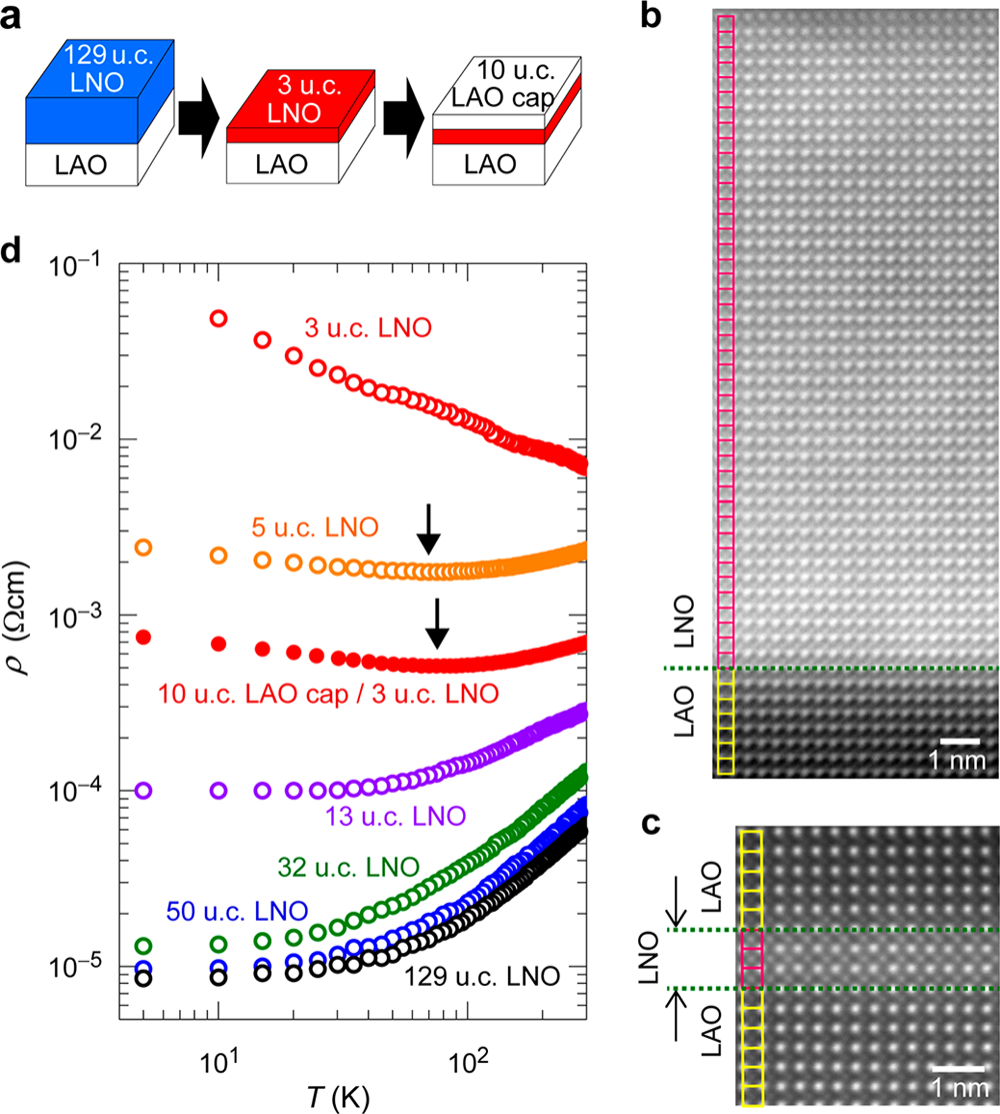
Heterostructure control and metal–insulator transition of LNO films on LAO substrates. (a) Schematic images of LNO thin-film heterostructures. (b) HAADF-STEM image of the heterointerface structure of a 50 u.c. LNO film/LAO substrate. The LNO film grows coherently with strain on the LAO substrate, where the clear atomic structure of the perovskite lattice is seen from the interface to the bulk region. (c) HAADF-STEM image for the trilayer structure of 10 u.c. LAO capping layer/3 u.c. LNO film/LAO substrate. The LAO/LNO/LAO heterostructure grows coherently with strain; i.e., the in-plane interatomic La–La distance remains constant from the substrate to the topmost capping layer and no dislocation is observed. (d) Temperature dependences of resistivity (*ρ*–*T*). The arrows indicate the metal-to-insulator crossover temperatures.

Figure 1d summarizes resistivity (*ρ*) vs temperature (*T*) curves for the LNO films. The *ρ* value at room temperature (RT) increases continuously as *t* is reduced to below 50 u.c. The thick LNO films with *t* ≥ 13 u.c. display metallic behaviors in the whole *T* range down to 5 K, while the thinner film with *t* = 5 u.c. shows a slight upturn in *ρ* below 70 K, and the ultrathin film with *t* = 3 u.c. shows insulating behavior in the whole *T* range, indicating a critical thickness for the metal-to-insulator (M-I) transition of ∼4 u.c. An additional 10 u.c. LAO capping layer drives the 3 u.c. LNO film to be more conductive again, showing a M–I transition at *T* = 77 K. This result indicates an increased Ni 3d–O 2p orbital overlap due to the suppressed polar distortion in the capped ultrathin LNO film.^15^

The *S*–*T* curves for the LNO films are shown in Figure 2a. All the films have negative *S*, indicating that electrons are the majority carriers. *S* is the sum of the electron diffusion part *S*_d_ and the phonon drag part *S*_g_. In metals within the free electron model, *S*_d_(*T*) follows the linear relation *S*_d_(*T*) = *AT*, where *A* is a constant proportional to m*n^–2/3^ (*n* is the carrier density).^23,24^ On the other hand for the LNO films, the observed *S*(*T*) curves are described well by a linear relation in the high-*T* region, though it has a finite *S*_d_(0) and is expressed by *S*_d_(*T*) = *S*_d_(0) + *AT*,^23^ as seen in Figure 2a and Figure S6b. The magnitude of the observed *S*(*T*) for each film has a maximum around *T* = 25–33 K, although there is no anomaly in the *ρ*(*T*) curves, which is ascribed to the phonon drag *S*_g_(*T*) and is not observed in polycrystalline bulk LNO (gray squares).^11^ Crucially, the *S*(*T*) peak value increases with decreasing *t* down to 3 u.c., and it is further enhanced by the LAO surface capping, resulting in a 10 times *S* enhancement over the bulk value. For thinner films, *S*(*T*) also becomes larger in a wide *T* range up to far above its peak temperature (e.g., 200 K for the LAO cap/3 u.c. LNO film), and the *S*(*T*) peak temperature shifts from 25 to 33 K.

**Figure 2:**
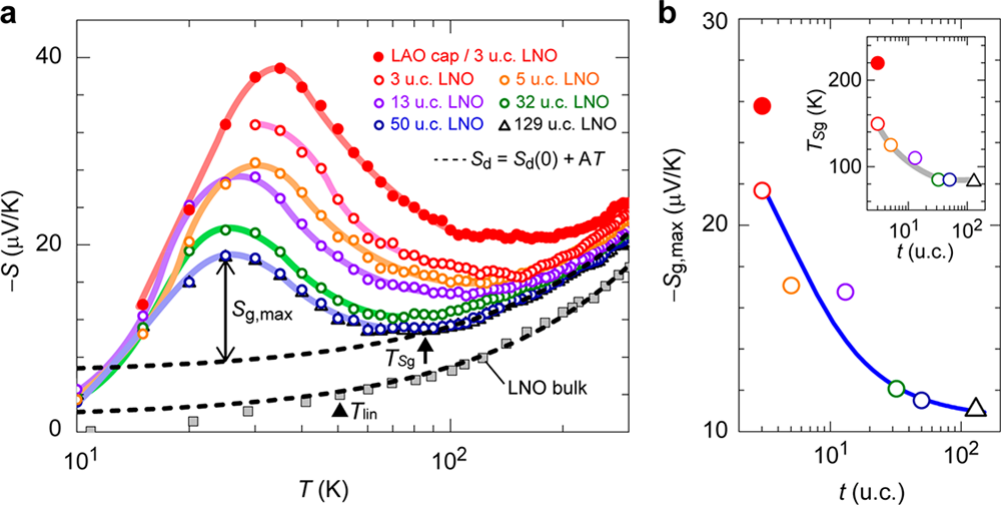
Thermopower (*S*) and phonon drag effect of LNO thin film heterostructures. (a) *S*–*T* for the uncapped LNO films with *t* ranging from 129 u.c. to 3 u.c. along with the LAO-capped 3 u.c. LNO film. The *S*–*T* curve of bulk LNO polycrystal (the gray squares)^11^ is shown for comparison. With decreasing *t*, the LNO films exhibit *S* enhancement, where the *S* value shows the maximum as a peak around *T* = 25–33 K and decreases rapidly with increasing *T* up to RT due to the phonon-drag effect (*S*_g_) contribution. The dashed lines indicate the linear *T* variations of electron diffusion *S*_d_(*T*) = *S*_d_(0) + *AT*. (b) *t* dependence of the maximum phonon-drag *S*_g_ value (*S*_g,max_), which is obtained from *S*_g_(*T*) *= S*(*T*) – (*S*_d_(0) + *AT*). The blue line indicates the fitting result using |*S*_g,max_(*t*)| = *A*(1 – e^*-t/l*_*p*_^)/*t* + |*S*_g,max_(*t* = ∞)|, where *l*_p_ is the phonon penetration depth of the leaked phonon, *A* is a proportional constant, and *S*_g,max_(*t* = ∞) is a constant corresponding to the *S*_g,max_ value of the strained LNO film with *t* = ∞. The inset shows the onset temperature *T*_*S*g_, where *S*_g_ starts to increase (indicated by the up arrow in (a)).

Here we should note that *S*(*T*) should approach zero when *T* decreases to absolute zero on the basis of thermodynamics. However, the experimentally obtained *S*_d_(0) values, including that of the LNO bulk, were all negative. These data suggest that |*S*_d_(*T*)| should decrease with a larger slope in the lower *T* region so that *S*_d_(*T*) will vanish at the absolute zero limit. It has been reported that strongly correlated electron systems exhibit nonlinear regimes in *S*_d_(*T*) with different slopes in the low-*T* region.^25,26^ In the LNO bulk, such a slope change is confirmed at *T*_lin_ ≈ 50 K,^11^ as seen by the deviations from the *S*_d_(*T*) = *S*_d_(0) + *AT* fitting at *T* < 50 K in Figure 2a and Figure S6c,d. In the LNO films in Figure 2a, it is difficult to determine *T*_lin_, as *T*_lin_ is located inside the phonon-drag-dominated regime. Therefore, we determined *T*_lin_ using strained LNO films without *S*_g_(*T*) peaks (the blue data in Figure S8a,b). Those films were grown at the lower *T* = 650 °C and have granular structures with ∼100 nm lateral sizes (Figure S7), which would cause phonon scattering at the grain boundaries and diminish the phonon-drag effect. This result suggests that the phonon mean free path is somewhere around 100 nm (see the Supporting Information for details). It should be noted that their *S*(*T*) curves show clear *T*_lin_ values at 45 K for the 5 and 50 uc LNO films, as shown by the triangles in Figure S8a,b. Then, we estimated *S*_d_(*T*) as *S*_d_(0) + *A*_HT_*T* for *T* > *T*_lin_ and *A*_LT_*T* for *T* < *T*_lin_, where *A*_HT_ and *A*_LT_ are the slopes of *S*(*T*) obtained above.

Then we estimated the phonon-drag contribution by *S*_g_(*T*) *= S*(*T*) – (*S*_d_(0) + *A*_HT_*T*). Since the true *S*_d_(*T*) value decreases more quickly at <*T*_lin_ as seen above, this *S*_g_(*T*) value provides a lower bound for the maximum *S*_g_ (*S*_g,max_) at 25–33 K. Figure 2b plots the *S*_g,max_ values at the peak temperature and *TS*g, the temperature where |*S*_g_| starts to increase (indicated by arrows in Figure S6a,b), as a function of *t*. When *t* is decreased to 3 u.c., |*S*_g,max_| is largely enhanced from 11 to 22 μV/K accompanied by a significant upward shift of *TS*g from 85 to 150 K (inset). The LAO capping further enhances |*S*_g,max_| to 26 μV/K, and *TS*g is pushed up to 220 K (the solid red circles), indicating that *S*_g_ contributes to the total *S* in a much wider *T* range in the presence of the LAO surface termination.

Next, we discuss the thickness dependences of m* on the basis of the temperature dependences of carrier transport properties. Figure 3a shows a longitudinal magnetoresistivity (MR), Δ*ρ*_*xx*_(*B*) = *ρ*_*xx*_(*B*) – *ρ*_*xx*_(0), where *ρ*_*xx*_(*B*) and *ρ*_*xx*_(0) are the resistivities with and without a magnetic field (*B*), respectively, from *T* = 4 to 100 K for the metallic uncapped LNO films with *t* = 50–13 u.c. In this *t* range, Δ*ρ*_*xx*_(*B*) values are positive and follow a quadratic field dependence. The magnitude of Δ*ρ*_*xx*_(*B*) becomes smaller with decreasing *t*. Note that even thinner films exhibit negative Δ*ρ*_*xx*_(*B*) values, as will be explained later. Figure 3b shows the transversal Hall resistivity *ρ*_*yx*_(*B*), where it is evident that Δ*ρ*_*yx*_(*B*) is positive for all *T*, manifesting a hole-dominated characteristic, and is the opposite sign of the electron-dominated *S*. LNO has a semimetallic electronic structure with the Fermi surface composed of Ni 3d and O 2p orbitals having electron and hole pockets;^27−29^ the respective electron and hole contributions would be the origin of the sign anomaly. Actually, the carrier concentration calculated by the single carrier model of the Hall effect as *n* = 1/(*q*|*R*_H_|) (*q* is the elementary charge and *R*_H_ the Hall coefficient) is 1.8 × 10^23^ cm^–3^ for the 50 u.c. LNO film (Figure 3c). This value is 10 times higher than the 1.8 × 10^22^ cm^–3^ obtained under the assumption that each trivalent Ni^3+^ (t_2g_^6^e_g_^1^) provides one election in LNO and is unreasonable. We therefore adopt a two-carrier model with one electron band and one hole band, providing the resistivity tensors, *ρ*_*xx*_ and *ρ*_*yx*_, by
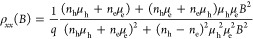
1
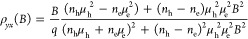
2where the suffixes e and h indicate electrons and holes, respectively.^30−32^ According to eq 2, the linear *ρ*_*yx*_(*B*) in Figure 3b indicates that electrons and holes are almost compensating each other, i.e. *n*_e_ ≈ *n*_h_ = *n* (Figure S10 supports the *n*_e_/*n*_h_ ratio ∼1.0, as it gives the best fit to the observed *ρ*_*yx*_(*B*)). Thus, we approximate eqs 1 and 2 by
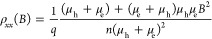
1′
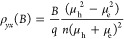
2′

**Figure 3:**
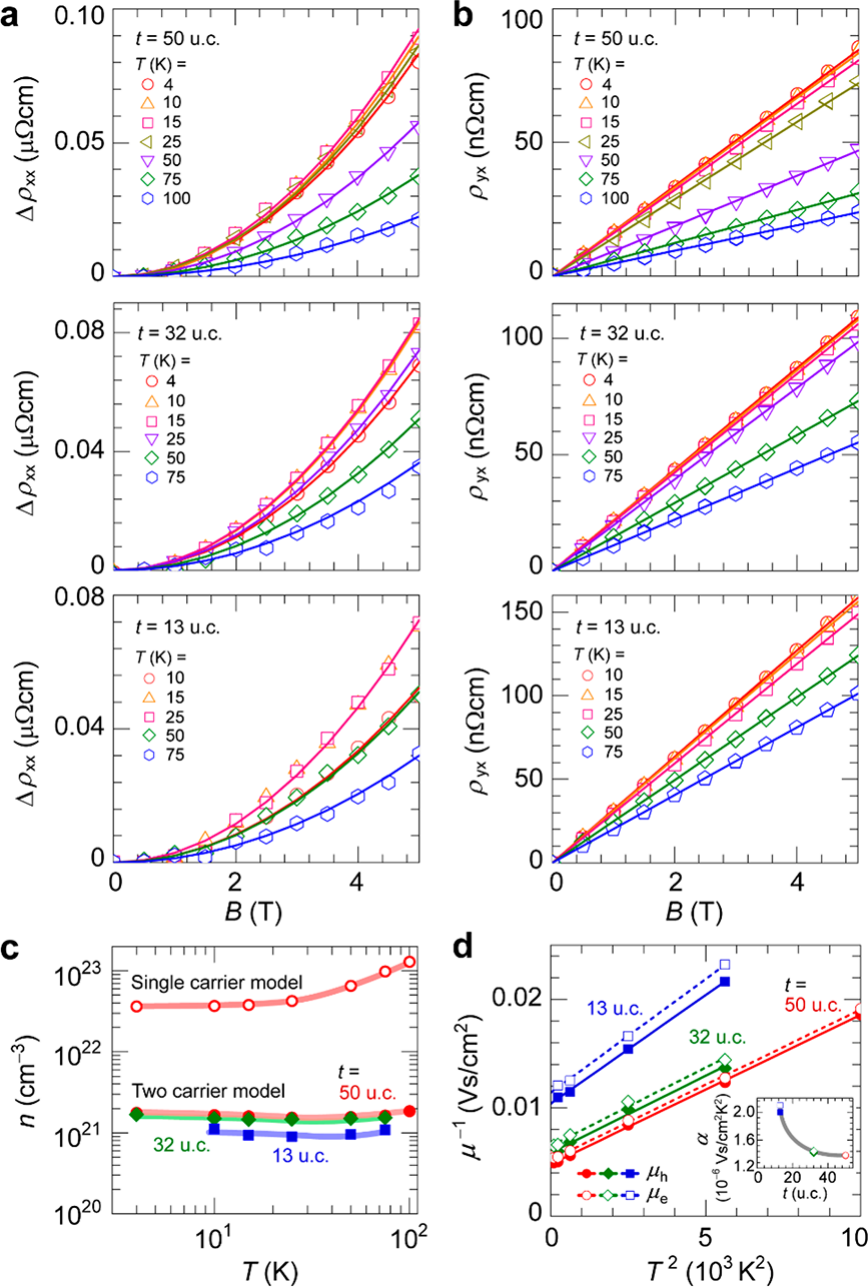
Two-carrier model analysis for carrier transport in metallic LNO films with *t* = 50–13 u.c. (a, b) Magnetic field (*B*) dependences of (a) longitudinal magnetoresistivity, Δ*ρ*_*xx*_(*B*) = *ρ*_*xx*_(*B*) – *ρ*_*xx*_(0) and (b) transversal Hall resistivity, *ρ*_*yx*_(*B*). The solid lines indicate the two-carrier model fitting. (c) Temperature dependence of carrier concentration, *n* (=*n*_e_ = *n*_h_), obtained by the two-carrier model. The *n* value obtained by a single-carrier model of the Hall effect for a 50 u.c. LNO film is also shown for comparison. (d) Temperature (*T*) dependences of the inverse of mobility (*μ*_e_, *μ*_h_) for the electron–electron scattering analysis. The inset shows the *t* dependence of the extracted parameter *α* from the fits to *μ*^–1^ = *αT*^2^.

The solid lines in Figure 3a,b show the fitting results to eqs 1′ and 2′, which reproduce the experimental results in the whole *B* region. The estimated *n* value is almost independent of *T* (Figure 3c), reflecting the metallic electronic structure, and decreases slightly for thinner films. On the basis of Matthiessen’s rule, the *μ*^–1^ value of metals shows a linear *T* dependence for e-ph scattering, but that of the LNO film does not follow such a linear dependence (Figure S12a), indicating that the contribution of e-ph scattering is not dominant. An electron–electron (e-e) scattering limited mobility in a Fermi liquid is described as , where *μ*_c_ is the Coulomb pseudopotential^33^ and *α* is a measure of the strength of e-e scattering.^34−36^Figure 3d shows *μ*^–1^ vs *T*^2^ plots for *μ*_e_ and *μ*_h_. The linear variations in the *μ*^–1^ vs *T*^2^ plots suggest that e-e scattering dominates for both electrons and holes in all of the films. The *μ*_e_/*μ*_h_ ratio decreases to ∼0.9 as *T* decreases and is almost independent of *t* (Figure S12b). The inset in Figure 3d shows the extracted *α* value as a function of *t*. It increases from 1.38 × 10^–6^ to 2.10 × 10^–6^ V s/(cm^2^ K^2^) for electrons and 1.37 × 10^-6^ to 2.00 × 10^–6^ V s/(cm^2^ K^2^) for holes as *t* decreases from 50 to 13 u.c. It gives *α*_13u.c._/*α*_50u.c._ = 1.52 for electrons and 1.46 for holes, respectively, indicating that the strength of e-e scattering is larger for thinner films. The effective mass ratio is given as m*_13u.c._/m*_50u.c._. Using the carrier concentration ratio of *n*_13u.c._^2/3^/*n*_50u.c._^2/3^ = 0.77 at 10 K, m*_13u.c._/m*_50u.c._ is estimated to be 1.08 for electrons and 1.06 for holes. The enhanced m* results in a decrease in *μ*_e_ and *μ*_h_ for thinner films. As explained above, the slope of *S*_d_(*T*), *A*_LT_, is proportional to m*n^–2/3^ at low *T*.^23,24^ Here we estimated *A*_LT_ from the strained LNO films with phonon-drag peaks at *t* ≥ 13 u.c., as plotted in Figure S6, where *T*_lin_ are taken from the strained LNO films without phonon-drag peaks in Figure S8a,b. This gives *A*_13u.c._/*A*_50u.c._ = 1.32 and m*_13u.c._/m*_50u.c._ ≈ 1.02, which is consistent with the above values obtained from the e-e scattering (1.08). These results support the conclusion that m* is enhanced with decreasing thickness for the metallic LNO films, but its magnitude is not large, although a mass renormalization with factors of 4–5 has been reported in an extremely thin insulating 1 u.c. LNO.^18^

We then investigated MR of thinner LNO films to clarify the dominant carrier scattering mechanisms for LNO films with and without the capping LAO layer. The insulating behavior of thin LNO films has been explained by the two-dimensional (2D) weak localization theory.^13^Figure 4a shows the *B* dependence of *σ*_s_(*B*) – *σ*_s_(0), where *σ*_s_(*B*) and *σ*_s_(0) are the sheet conductances with and without *B*, respectively, for the uncapped 5 u.c. LNO films and the LAO-capped 3 u.c. LNO film. The difference *σ*_s_(*B*) – *σ*_s_(0) increases with *B* and becomes large in the low-*T* region. Note that the measured *σ*_s_(*B*) data were not reliable for the uncapped 3 u.c. LNO film in the low-*T* region because of its high *ρ* value; therefore, we compare the uncapped 5 u.c. LNO film and the LAO capped 3 u.c. LNO film. For the 2D weak localization regime at low *T*,^37^ in the absence of spin-flip scattering, *σ*_s_(*B*) can be expressed as , where *ψ*(*x*) is the digamma function and *L*_in_ is the inelastic scattering length of electrons which scales with the *T* as *L*_in_ = *BT*^–*p*/2^. The *p* value depends on the inelastic scattering mechanism; e.g., *p* = 1 for e-e scattering, and *p* = 3 for e-ph scattering. The fitting results shown by the solid lines in Figure 4a sufficiently explain the experimental *σ*_s_(*B*) curves. The extracted *L*_in_ value shown in Figure 4b exhibits almost the same *T* variation as in the above model for *p* = 1, indicating that e-e scattering dominates at *T* ≤ 15 K. The estimated *L*_in_ value at 15 K is 4.5 nm (∼12 u.c.), which is much greater than the film thickness, hence indicating that these films are indeed in the 2D transport regime. Both of the films show an upturn in *ρ* at *T* < 70 K (Figure 1d), which is a characteristic of a 2D metal with finite disorder.^38,39^ For this localized regime, electrons hop between localized states, and *σ*_s_ can be approximated by , where *σ*_0_ is the dc Drude conductivity, *T*_0_ is related to the transport mean free path, and *p* comes from *L*_in_ ≈ *T*^–*p*/2^.^39^Figure 4c shows *σ*_s_–ln *T* plots, where *σ*_s_ is normalized by e^2^/*πh* ≈ 1.1 × 10^–5^ S. The uncapped 5 u.c. LNO film shows a linear ln(*T*) variation with *p* = 1, while the LAO-capped 3 u.c. LNO film shows a linear ln(*T*) variation with *p* = 1 for *T* < 15 K and *p* = 3 for *T* = 20–40 K, indicating that the LAO surface termination of the thin LNO film enhances the e-ph scattering. As stated above, in , *f* is the fraction of the crystal momentum that is transferred from the phonons to the electrons. Since , the enhanced e-ph scattering contributes to the enhanced *S*_g_. As a paramount ingredient of the phonon-drag *S*_g_, this capping-induced boost in the effective e-ph scattering emerges as the most likely driver of the significant enhancement of the *S* in the same *T* range.

**Figure 4:**
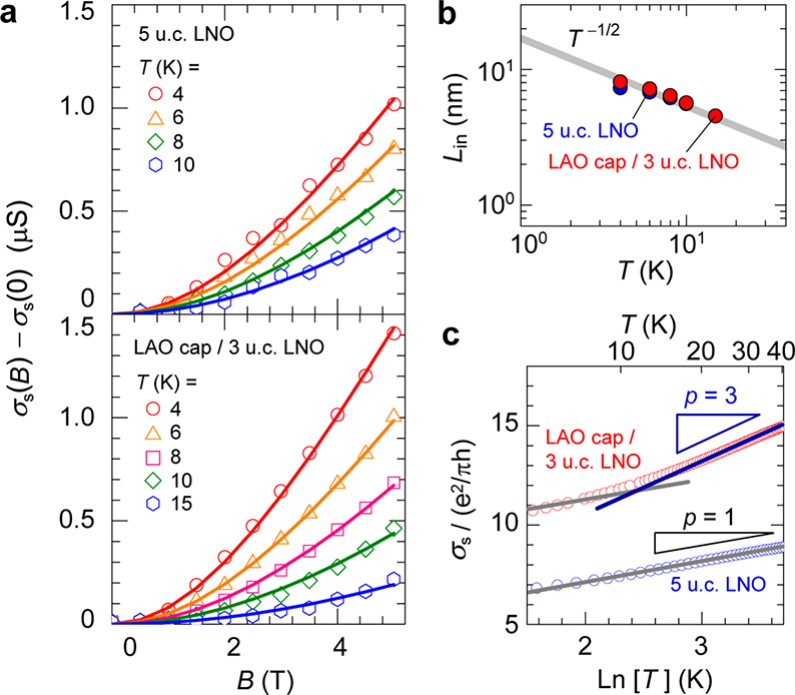
A 2D weak localization model analysis for carrier transport of an LNO ultrathin film and heterostructure. (a) Magnetic field (*B*) dependence of sheet conductance (*σ*_s_(*B*) – *σ*_s_(0)) for a 5 u.c. LNO film (top panel) and an LAO-capped 3 u.c. LNO film (bottom panel) at *T* = 4–15 K. The solid lines indicate the fitting curve to the 2D weak-localization model. (b) Extracted inelastic scattering length (*L*_in_) as a function of *T*. The solid line indicates the *T*^–1/2^ dependence. (c) *σ*_s_ as a function of ln(*T*) in the weakly insulating range *T* < 40 K. The solid lines indicate the slope of the linear fits, providing *p* in .

As seen in Figure 2a, the *S*_g_ peak temperature of the LNO film was enhanced from ∼25 to ∼33 K by the reduction of the film thickness and the surface termination, and the enhanced peak temperature 33 K is close to the peak temperature of ∼40 K in the thermal conductivity (κ) of LNO^40^ and LAO^41^ (Figure S13). This coincidence suggests that the observed *S*_g_(*T*) value is dragged by phonons leaking from the LAO substrate and the capping layer. We therefore analyze the phonon penetration depth of the leaked phonon *l*_p_ in the LNO films following the method of Wang et al.^21^ They proposed that the local phonon-leaking *S* at depth *x*, *S*_g,max_(*x*), is proportional to the flux of the leaking phonon, *F*(*x*) = *F*_0_*e*^–*x/l*_p_^, and the observed *S*_g,max_(*t*) for a film with the thickness *t* is the average value of the integrated *S*_g,max_(*x*), giving |*S*_g,max_(*t*)| = *A*(1 – *e*^–*t/l*_p_^)/*t* + |*S*_max_(*t* = ∞)|, where *A* is a proportional constant and *S*_max_(*t* = ∞) is a constant corresponding to the *S*_g,max_ of a strained LNO film with *t* = ∞. The fitting result is superimposed on Figure 2b, and we obtained *l*_p_ ≈ 1 nm. This *l*_p_ value is 10 times longer than that of 0.1 nm in the Bi_2_Te_3_/Al_2_O_3_ interface.^21^ In the present case, LNO and LAO have the same perovskite crystal structure with the same mass elements, La and O, forming a similar phonon band structure and allowing for the larger *l*_p_. These results suggest that an e-ph coupling between the charge carriers in the LNO thin film and the phonons with a long mean free path of ∼100 nm is further enhanced by phonon leakage from the substrate/capping layer. The present results demonstrate that in atomic-scale controlled heterostructures of strongly correlated TMOs it is possible to manipulate both the electronic and phononic properties in the quest for high-performance thermoelectrics.

## Supporting Information Available

X-ray reciprocal space map, field-emission scanning Auger electron spectra, X-ray absorption spectra, electron energy loss spectra, hard X-ray photoemission spectra, phonon-drag thermopower analysis, two-carrier model analysis for Hall resistivities and magnetoresistivities, carrier transport analysis of LaNiO_3_ films and heterostructures, and thermal conductivity of LaAlO_3_ and LaNiO_3_ (PDF)
