# Supplementary material for: Large phonon drag thermopower boosted by massive electrons and phonon leaking in LaAlO3/LaNiO3/LaAlO3 heterostructure
Source: Nano Lett. 2021 Oct 28;21(21):9240–6. doi: 10.1021/acs.nanolett.1c03143 (PMC8587880; doi:10.1021/acs.nanolett.1c03143)
Supplement: Supplementary file 1 — nl1c03143_si_001.pdf [file nl1c03143_si_001.pdf]

Supporting Information

Large phonon drag thermopower boosted by massive electrons and phonon leaking in $LaAlO_3$ / $LaNiO_3$ / $LaAlO_3$ heterostructure

Masatoshi Kimura[1], Xinyi He[1], Takayoshi Katase[1,2], Terumasa Tadano[3], Jan M. Tomczak[4], Makoto Minohara[5], Ryotaro Aso[6], Hideto Yoshida[7], Keisuke Ide[1], Shigenori Ueda[8,9,10], Hidenori Hiramatsu[1,11], Hiroshi Kumigashira[12,13], Hideo Hosono[11], and Toshio Kamiya[1,11*]*

[1] Laboratory for Materials and Structures, Institute of Innovative Research, Tokyo Institute of Technology, 4259 Nagatsuta, Midori, Yokohama 226-8503, Japan

[2] PRESTO, Japan Science and Technology Agency, 7 Gobancho, Chiyoda, Tokyo, 102-0076, Japan

[3] National Institute for Materials Science, Sengen, Tsukuba 305-0047, Japan

[4] Institute of Solid State Physics, Vienna University of Technology, Wiedner Hauptstrasse 8-10, A-1040 Vienna, Austria

[5] Research Institute for Advanced Electronics and Photonics, National Institute of Advanced Industrial Science and Technology, Tsukuba, Ibaraki 305-8568, Japan

[6] Department of Applied Quantum Physics and Nuclear Engineering, Kyushu University, Fukuoka, Fukuoka 819-0395, Japan

[7] The Institute of Scientific and Industrial Research, Osaka University, 8-1 Mihogaoka, Ibaraki, Osaka 567-0047, Japan

[8] Research Center for Functional Materials, National Institute for Materials Science, Namiki, Tsukuba 305-0044, Japan

[9] Research Center for Advanced Measurement and Characterization, National Institute for Materials Science, Tsukuba 305-0047, Japan

[10] Synchrotron X-ray Station at SPring-8, National Institute for Materials Science, 1-1-1 Sayo, Hyogo, 679-5148, Japan

[11] Materials Research Center for Element Strategy, Tokyo Institute of Technology, 4259 Nagatsuta, Midori, Yokohama 226-8503, Japan

[12] Photon Factory, Institute of Materials Structure Science, High Energy Accelerator Research Organization, Tsukuba, Ibaraki 305-0801, Japan

[13] Institute of Multidisciplinary Research for Advanced Materials, Tohoku University, Sendai 980-8577, Japan

*Corresponding Author: katase@mces.titech.ac.jp, kamiya.t.aa@m.titech.ac.jp

Contents

Materials and Methods

Film structure, chemical composition, and electronic state analyses of $LaNiO_3$ film

Phonon drag thermopower analysis of $LaNiO_3$ film

Two carrier model analysis for Hall and Magneto resistivities of $LaNiO_3$ film

Carrier transport analysis of $LaNiO_3$ film

Thermal conductivity of $LaAlO_3$ and $LaNiO_3$

Materials and methods

Thin film growth. $LaNiO_3$ (LNO) epitaxial films were grown on a pseudo-perovskite substrate of (001) $LaAlO_3$ (LAO) with $\Delta a/a$ (= $(a_{LAO} - a_{LNO}) / a_{LNO} \times 100$) of −1.5%, where a_{LAO} and a_{LNO} are pseudo-cubic lattice constants of LAO substrate (3.78 Å) and LNO bulk (3.8377 Å).[1] A KrF excimer laser (wavelength of 248 nm) was used to ablate a polycrystalline target disk consisted of La_2NiO_4 and NiO phases, where the laser energy fluence and the repetition rate were 0.5 J cm^{-2} and 2 Hz, respectively. Prior to the film growth, LAO substrate was chemically etched by HCl solution and annealed in a vacuum at 700 °C to obtain a step-and-terrace surface. The growth temperature was fixed at 700 °C and O_2 pressure was optimized to 25 Pa. A 10 u.c. LAO capping layer is deposited at 500 °C at P_{O2} = 25 Pa. After the deposition, the substrate temperature is decreased down to room temperature (RT) in the same O_2 pressure, and then the sample was annealed in O_2 1atm for 1 hour to ensure oxygen stoichiometry.

Electronic property measurement. The resistivity (ρ) was measured by d.c. four probe method in the van der Pauw electrode configuration. The magneto-resistivity (ρ_{xx}) and Hall resistivity (ρ_{yx}) were measured using a 6-terminal Hall-bar structure under external magnetic fields of up to 5 T applied parallel to the out-of-plane direction and at the DC-current in the in-plane of the epitaxial film. The thermopower (S) was measured by giving a temperature difference (ΔT) up to 2–4 K along the in-plane direction. The thermo-electromotive force (ΔV) and ΔT were simultaneously measured, and the S was obtained from the slope of the ΔV–ΔT plots, where the experimental error is estimated to be < 0.6 μV/K from the deviations of the ΔV–ΔT data.

Film structure and electronic state analyses. The out-of-plane and in-plane lattice constants of LNO films were measured via reciprocal space mapping (RSM) by high-resolution X-ray diffraction (XRD, anode radiation: monochromatic CuKα_1) at RT. The film surface structures were observed by atomic force microscopy (AFM). Cross-sectional thin-film sample for scanning transmission electron microscopy (STEM) observation was prepared by focused ion

beam (FIB) and Ar^+ ion milling. The cross-sectional microstructure was examined by high-resolution STEM (JEM-ARM200F, JEOL Ltd.), where the electron incident direction was parallel to LAO [100]. For the LAO-capped LNO film, the Ni^{3+} state was also confirmed by electron energy loss spectroscopy (EELS) spectra measured by Continuum K3-IS (Gatan) attached to Titan ETEM (Thermo Fisher Scientific) under the O_2 pressure of 20 Pa.[2] The homogeneous chemical compositions in LNO film / LAO substrate were confirmed by field-emission scanning Auger electron spectroscopy (FE-AES) along the depth direction. In FE-AES measurements, the incident electron beam was weakly accelerated at 3 kV and 10 nA to suppress the electrical charge-up of the specimens. The depth profiles were established through Ar^+ ion sputtering of the films. The stoichiometric Ni^{3+} oxidation state with an unnoticeable amount of oxygen deficiencies in the un-capped and the LAO capped LNO films was confirmed by X-ray absorption spectroscopy (XAS) measurements at the undulator beamline BL-2A of Photon Factory, high Energy Accelerators Research Organization (KEK), where the XAS spectra were taken in the total electron yield mode. The Hard X-ray photoemission spectroscopy (HAXPES) measurements with the excitation photon energy of 5.95 keV were conducted at the undulator beamline BL15XU of SPring-8. We confirm the chemical homogeneity and dominant Ni^{3+} valance state, which guarantee the investigation of the intrinsic thickness dependence and surface termination effects on carrier transport properties of LNO films.

Density functional theory calculation. The calculation of lattice thermal conductivity of LNO was carried out by using the Peierls–Boltzmann theory (PBT) within the relaxation-time approximation, as implemented in the ALAMODE code.[3] The phonon frequency and group velocity $\boldsymbol{v}_\lambda$ were calculated from the second-order force constants obtained by using density functional perturbation theory, as implemented in Quantum ESPRESSO package,[4] for which we employed $4 \times 4 \times 4$ q point mesh. The third-order anharmonic force constants were also calculated by using the finite-displacement method with the $2 \times 2 \times 2$ supercell. These density functional theory (DFT) calculations were conducted within the generalized gradient

approximation (GGA) parameterized by Perdew–Burke–Ernzerhof (PBE).[5] From the calculated force constants, the intrinsic phonon-phonon scattering rate $\tau_{q,\mathrm{p-p}}^{-1}$ was calculated. For the PBT calculation, the total scattering rate defined as $\tau_q^{-1} = \tau_{q,\mathrm{p-p}}^{-1} + \tau_{q,\mathrm{p-b}}^{-1}$ was used to incorporate the effect of grain boundaries, where $\tau_{q,\mathrm{p-b}}^{-1} = 2|\boldsymbol{v}_q|/L$ is the phonon-boundary scattering rate with L being the grain size. The PBT calculation was conducted with $20 \times 20 \times 20$ q point mesh, with which we obtained converged results.

Film structure, chemical composition, and electronic state analyses of $LaNiO_3$ films

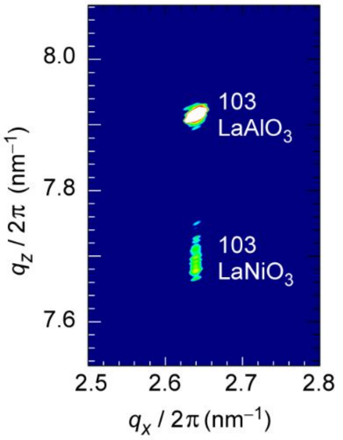

Figure S1. X-ray reciprocal space map (RSM) for 50 u.c. LNO film grown on LAO substrate. The RSM around the 103 reflection in pseudo-cubic perovskite unit cell with Pendellösung interference fringes substantiates the coherent epitaxy of LNO film on LAO substrate. The lattice constants for out-of-plane (c) and in-plane (a) are 3.88 Å and 3.78 Å, respectively. The lattice constant ratio (c/a) is 1.026, indicating the in-plane compressive strain in the LNO film.

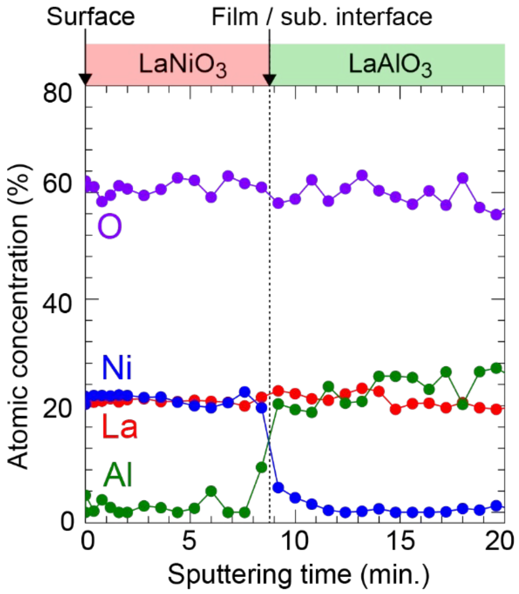

Figure S2. Field-emission scanning Auger electron spectroscopy (FE-AES) spectra for 50 u.c. LNO film on LAO substrate. The atomic concentration for the constituents of LNO and LAO was estimated from the signal intensity of Auger electrons. The positions of the film surface and the film/substrate interface are indicated at the top of the panel. In the bulk region from surface, the elemental distributions of LNO film appear to be homogeneous and La/Ni ratio is

constant within the accuracy of the FE-AES measurements. The atomic concentrations of Ni and Al sharply decrease and increase at the film/substrate interface, respectively.

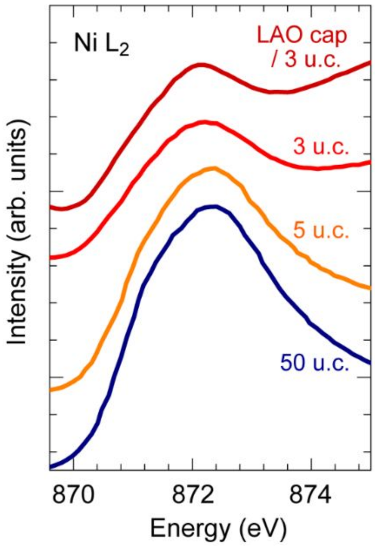

Figure S3. X-ray absorption spectra (XAS) of Ni L_2 edge for un-capped LNO films with thicknesses of 3 u.c. (red line, '3 u.c.'), 5 u.c. (orange line,'5 u.c.'), and 50 u.c. (blue line, '50 u.c.'), as well as LAO-capped LNO film with 3 u.c. (dark red line, 'LAO cap / 3 u.c.') on LAO substrates at 25 K. The peak position of XAS spectra is independent on the thickness. The XAS spectra cannot be explained by a single peak of Ni^{3+} $3d^7$ configuration and there is a weak shoulder structure at lower energy side around 871.8 eV; this peak character is explained by a coherent mixture of Ni $3d^8$ and $3d^8L^2$ configuration, where L denotes a hole on an oxygen ligand.[6-8] Since it is known that Ni^{2+} of NiO has clearly separated two peaks at 870.2 eV and 871.2 eV, the present results support the stoichiometric Ni^{3+} valence state in all the LNO films and excludes the possible contribution of oxygen off stoichiometry to electronic properties.[9]

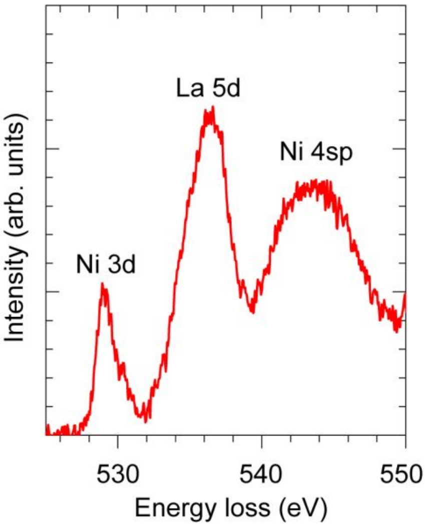

Figure S4. Electron energy loss spectra (EELS) for LNO layer in LAO-capped 3 u.c. LNO film, taken by Titan ETEM under O_2 pressure of 20 Pa at RT. The clear Ni 3d peak with charge state 3+ is seen at around 529 eV, which is consistent with previous report,[10] where the oxygen reduced $LaNiO_{2.75}$ and $LaNiO_{2.57}$ films showed clearly separated two peaks at 528.5 eV and 530.5 eV.

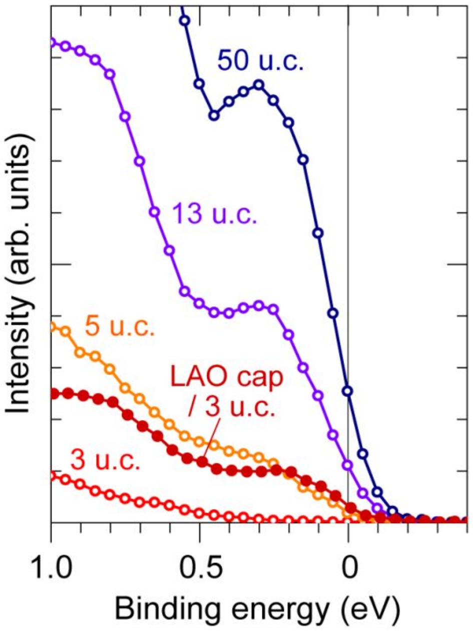

Figure S5. Hard x-ray photoemission spectra (HAXPES) normalized by deep La 5p peaks for the un-capped LNO films with LNO thicknesses of 50 u.c., 13 u.c., 5 u.c., 3 u.c, and LAO-capped LNO film with 3 u.c. at RT. We confirm the metal-insulator transition, where the spectral intensity around the Fermi level becomes smaller with decreasing *t* and becomes almost zero at $t = 3$ u.c, while it recovers by the LAO surface capping. Note that valence band of LAO does not contribute to this energy region because of large bandgap.[11]

Phonon-drag thermopower analysis of $LaNiO_3$ films

Figure S6 summarizes the $S(T)$ curves for the un-capped LNO films with different t and the LAO-capped 3 u.c. LNO film as well as those of LNO bulk polycrystals. In order to discuss the contribution of phonon-drag S_g to the total S of the LNO films, we measured the $S(T)$ curves of LNO films without S_g as a reference to obtain the T dependence of the diffusion contribution S_d. For this, we fabricated the 5 u.c. and 50 u.c. LNO epitaxial films with granular structures (grain size ~100 nm) on LAO substrates at lower growth temperature of 650 °C. **Figures S7a,b** compare the film surface morphology and the ρ–T curves for the 50 u.c. LNO films grown at 700 °C and 650 °C. The 650 °C grown LNO film with granular structures shows a slightly higher ρ than that of 700 °C grown LNO film with a step & terrace surface presumably due to grain boundary scattering. The S–T curves of 5 u.c. and 50 u.c. LNO films are summarized in **Figure S8**. The S peak at low T disappeared for the 650 °C grown LNO films with the grain sizes of ~100 nm. This result is corroborated by the lattice thermal conductivity (**Figure S9**), calculated by the Peierls–Boltzmann theory within the relaxation time approximation as implemented in the ALAMODE code,[3] which is largely decreased by the formation of the grain boundaries, supporting that the increase of S at low T can be ascribed to the phonon-drag S_g. Note that the S of 5 u.c. LNO film with granular structures is suppressed even at high T ~100 K, compared to that of 5 u.c. LNO film with a step & terrace surface. This is consistent with that the formation of 100 nm size grain structure largely suppress the lattice thermal conductivity at T up to 200 K (**Figure S9**). These results suggest that the phonons with long mean free path > 100 nm could have a strong correlation with the electrons in LNO films to induce the phonon-drag S_g. In addition, the total S is largely increased by the S_g contribution in wider T range for thinner LNO films.

In $S(T)$ curves of all LNO films (**Figure S6**), we here assume a single linear relation $S_d(T) = S_d(0) + AT$ that reproduces the experimental result accurately in the high T region (see dashed lines). Note that LNO has a semi-metallic electronic structure with the Fermi surface composed

of Ni 3d and O 2p orbitals having electron and hole pockets.[15-17] Therefore, the analysis of S_d should consider a two carrier model; the two carrier model for S_d is not always following the linear relation because it is expressed as $S_{e+h} = \frac{S_h - \frac{\sigma_e}{\sigma_h}|S_e|}{1 + \frac{\sigma_e}{\sigma_h}}$ (σ denote electronic conductivities, and the subscripts e and h denote those of electrons and holes, respectively).[18] However, we can approximately apply the linear temperature dependence for the electron – hole mixed conduction case (two carrier model) for our case by the following two confirmations. First, we calculated the T dependence of S_d for LNO by first-principles density functional theory codes, VASP and BoltzTraP2, based on full-band Bolzmann transport theory. We confirm the linear T dependence in the $S_d(T)$ plots along in-plane xx-axis and yy-axis, where Fermi level (E_F) does not shift largely. Second, assuming σ_e and σ_h have similar temperature dependences, which is supported by similar temperature dependences of electron and hole mobilities extracted from the two carrier model in Fig. 3d, and thus $\frac{\sigma_e}{\sigma_h} \sim r_\sigma$ (constant), $S_{e+h}$is approximated to follow a linear relation $\frac{A_h - r_\sigma A_e}{1 + r_\sigma}T$ ($S_h = A_h T$, $S_e = A_e T$). However, the S_d fit leads to a finite intercept $S_d(0)$; the non-zero intercept $S_d(0)$ is not physical but an artifact from the linear fit at high T region. The $S_d(T)$ should approach to 0 when T decreases to 0 in the free electron model. Actually, the true S_d linearly goes to 0 at T less than $T_{lin} = 45$ K (see the deviation between the fitted dashed line and the experimental data for $S(T)$ curves of LNO bulk and the low-T grown LNO films with granular structures in **Figure S6 and S8**). In order to estimate the S_g contribution to total S exactly, it is required to explain the T dependence of S_d perfectly at all T stage. However, since the true $S_d(T)$ decreases at T less than $T_{lin} = 45$ K, we only claim that the $S_g(T) = S(T) - (S_d(0) + AT)$ provides a lower bound for the $S_{g,max}$ at the S peak temperature 25–33 K of LNO films in this paper.

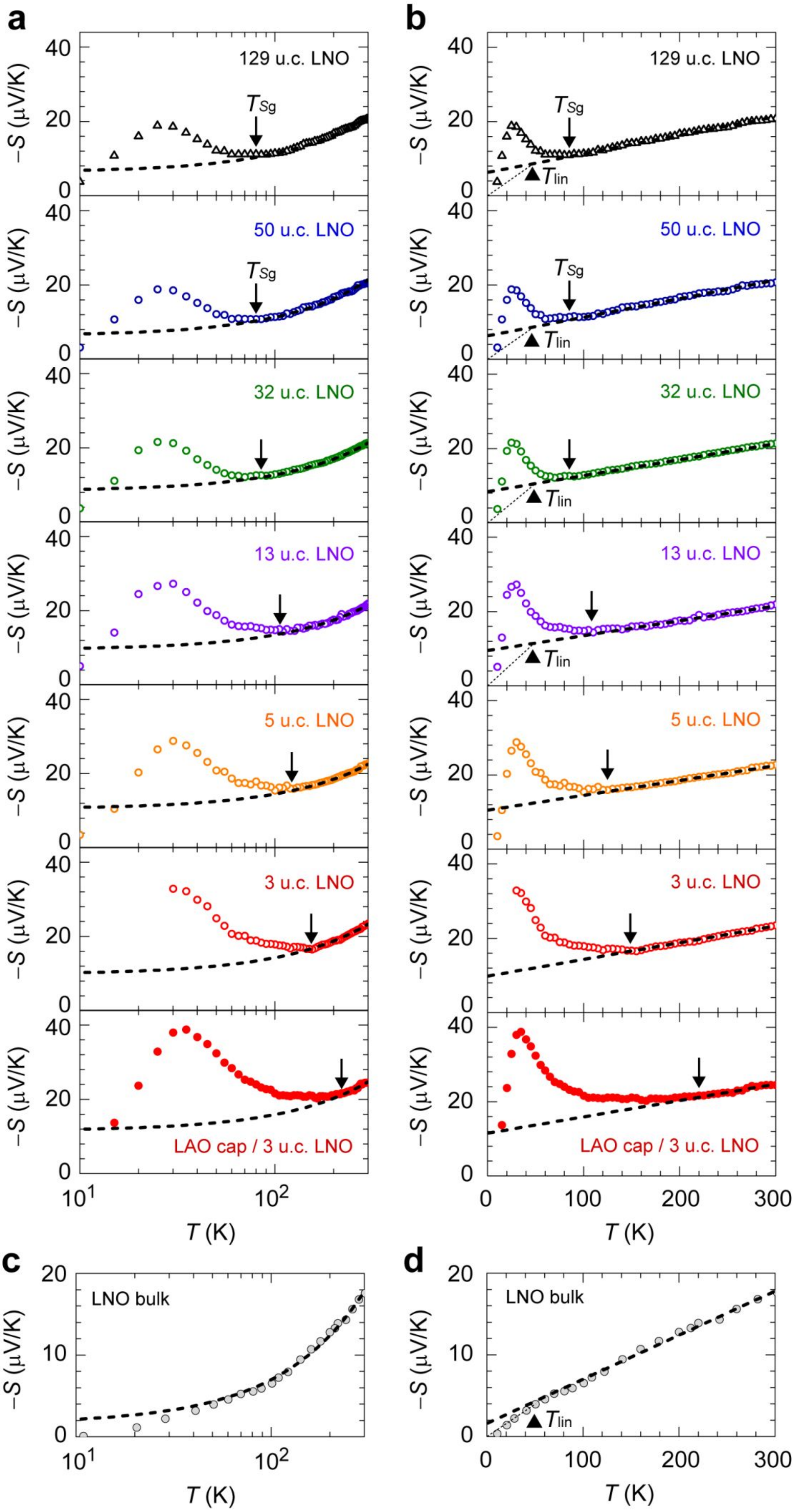

a
b
c
d
129 u.c. LNO
50 u.c. LNO
32 u.c. LNO
13 u.c. LNO
5 u.c. LNO
3 u.c. LNO
LAO cap / 3 u.c. LNO
LNO bulk
T_{Sg}
T_{lin}
−S (μV/K)
T (K)

Figure S6. Temperature (T) dependences of thermopower (S) for LNO films with different t and LAO-capped 3 u.c. LNO film. (a) S vs. T with logarithmic scale. (b) S vs. T with linear scale. (c,d) The S–T curves for bulk LNO are shown for comparison. The dashed lines indicate the T variation of $S_d(T) = S_d(0) + AT$. The T_{Sg}, where the S_g starts to increase, is indicated by the arrows. The upward triangles indicate the temperature (T_{lin}) where $S(T)$ changes its slope from the linear fitting of $S_d(T)$.

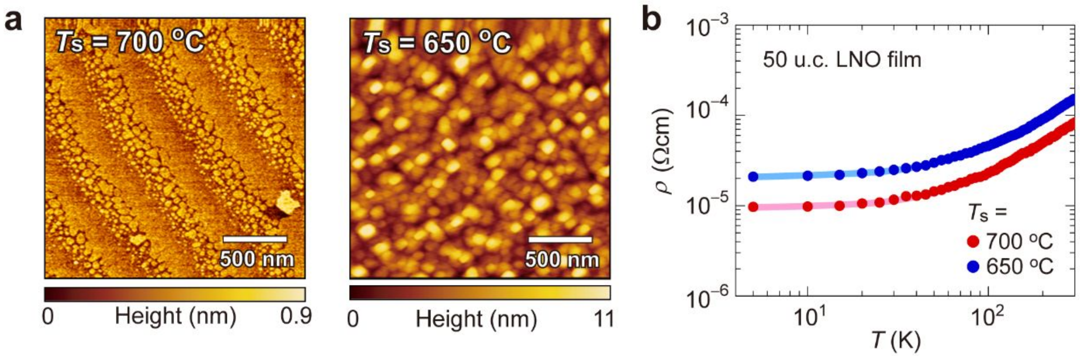

Figure S7. (a) Atomic force microscopy images and (b) temperature (T) dependence of resistivity (ρ) for 50 u.c. LNO film grown at optimized substrate temperature T_s = 700 °C and reduced T_s = 650 °C.

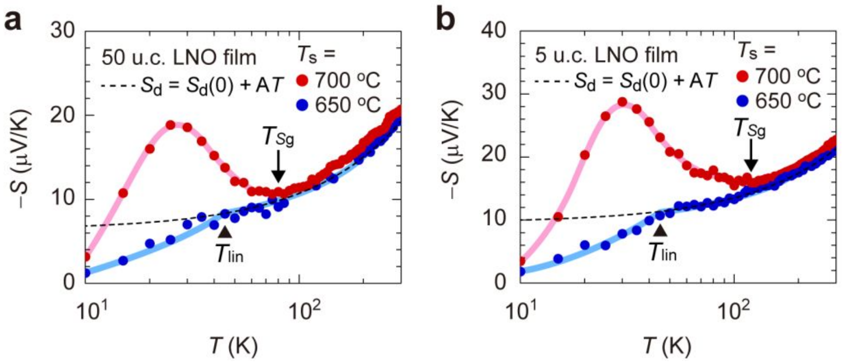

Figure S8. Temperature (T) dependences of the thermopower (S) for (a) 50 u.c. and (b) 5 u.c. LNO films with granular structures (grain size ~100 nm) grown at T = 650 °C. In (b) and (c), the S–T curves for high-quality LNO films with a step & terrace surfaces grown at T = 700 °C are shown for comparison. The dashed line indicates the linear temperature variation of $S_d(T)$ = $S_d(0) + A_{HT}T$ fitted to the high T regime. The downward triangles indicate the temperature (T_{lin}) where the S starts to decrease from the linear fitting of $S_d(T)$ for LNO films with granular structures. These data suggest that the phonon drag S_g can be suppressed by grain boundaries, revealing (to good approximation) the purely diffuse S_d contribution.

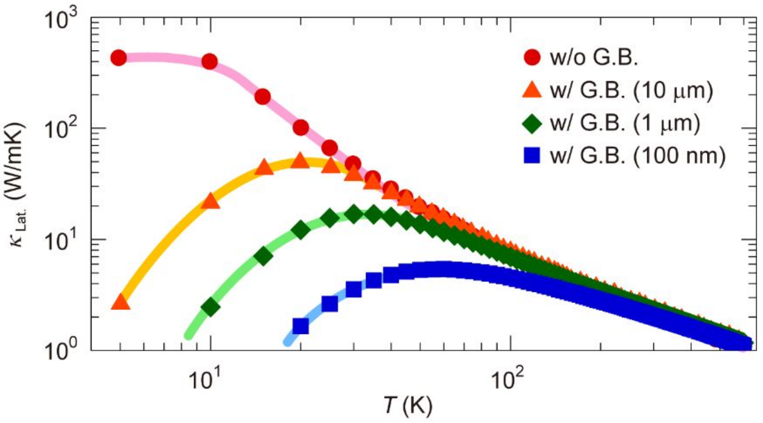

Figure S9. Calculated lattice thermal conductivity ($\kappa_{\mathrm{Lat.}}$) for LNO without grain boundary (G.B.) and with 10-μm, 1-μm, 100-nm G.Bs.

Two carrier model analysis for Hall and Magneto resistivities of $LaNiO_3$ films

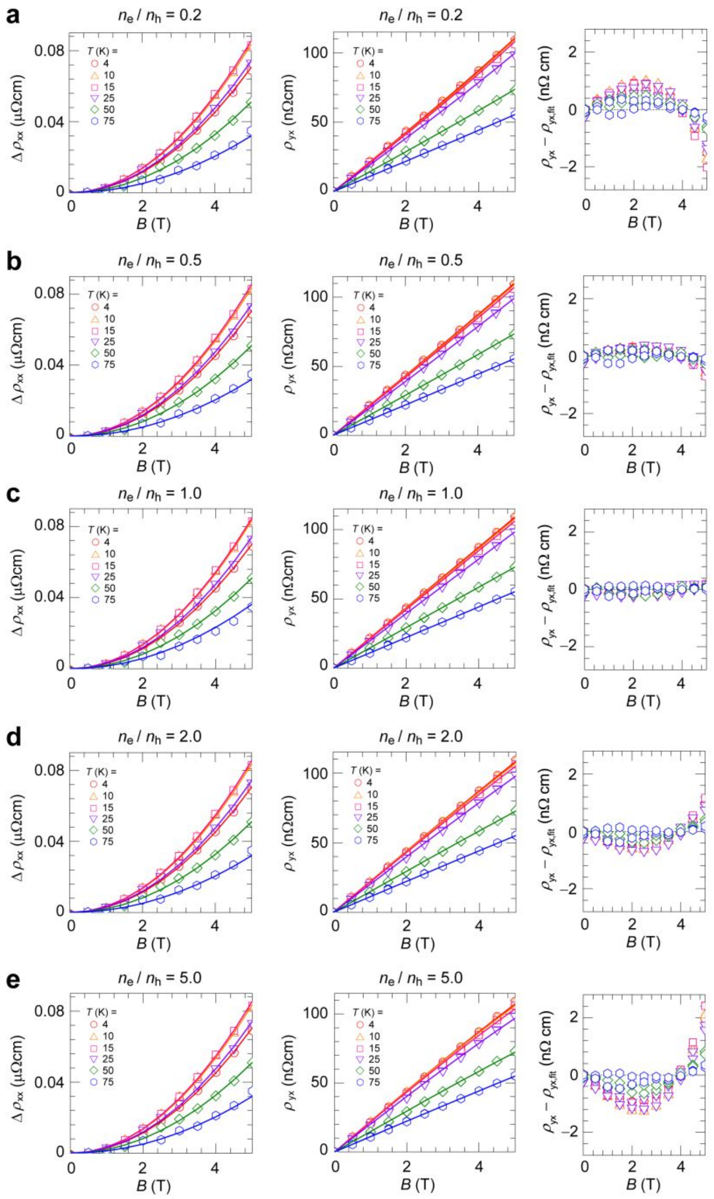

Figure S10. Two carrier model analysis for carrier transport in LNO films with t = 32 u.c. by considering the different electron and hole concentration ratio, i.e. n_e/n_h = 0.2 (a), 0.5 (b), 1.0 (c), 2.0 (d), and 5.0 (e). (Left) Magnetic field (B) dependence of longitudinal magneto-resistivity (MR), $\Delta\rho_{xx}(B) = \rho_{xx}(B) - \rho_{xx}(0)$, and (Middle) B dependence of transverse Hall resistivity, $\rho_{yx}(B)$, measured at different temperatures of 4–75 K. The solid lines indicate the two carrier model fitting. (Right) The difference between experimental ρ_{yx} and fitting result $\rho_{yx,fit}$, i.e. $\rho_{yx} - \rho_{yx,fit}$. Although the longitudinal MR $\rho_{xx}(B)$ can be fitted well by optimizing the μ_e and μ_h (left panels), deviations are seen in the transversal MR (Hall) $\rho_{yx}(B)$ fitting result to the experimental $\rho_{yx}(B)$ in high magnetic field regions (the middle and the right panels, respectively), The deviation becomes larger if the n_e/n_h goes away from 1.0. These results support that n_e/n_h = 1 is most appropriate.

Carrier transport analysis of $LaNiO_3$ films

The effect of electronic correlations on transport properties of metallic LNO bulk and thin films has been discussed in 19,20. It is known that the ρ–T curves at $T < 80$ K of LNO bulk and thin film show a quadratic T dependence, $\rho = \rho_0 + AT^2$, which is a characteristic of electron-electron (e-e) interactions based on Landau's Fermi liquid.[21] The ρ_0 is residual resistivity and A is a measure of the strength of e-e scattering. We also confirmed that ρ–T curves of metallic LNO films with t = 50, 32, 13 u.c. follow the power low of $\rho = \rho_0 + AT^2$, as shown in **Figure S11a**. This is also consistent with the T^2 dependence of μ^{-1}, while the n has almost no T dependence. The estimated ρ_0 and A values are summarized in **Figures S11b,c**. The A value increases with decreasing t, supporting the strong e-e scattering in thinner LNO films at low T.

We calculated the electron and hole mobility ratio, μ_e/μ_h for metallic LNO films with t = 13 u.c., 32 u.c., and 50 u.c. from the result of the two carrier model analysis. The μ_e/μ_h decreases down to ~0.9 at low T (**Figure 12b**). This result suggests that the negative S_d originate from the lower μ_e than μ_h of LNO films.

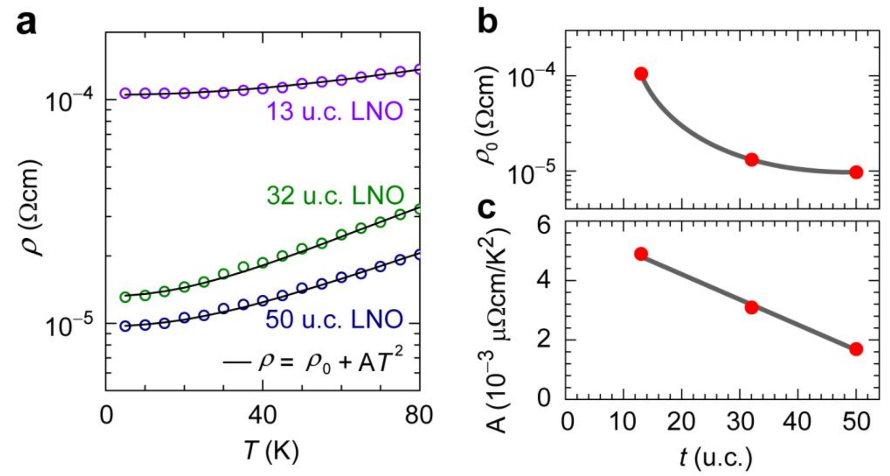

Figure S11. (a) Temperature (T) dependence of the resistivity (ρ) for 50−13 u.c. LNO films at $T \leq 80$ K. The solid lines indicate the fitting result using $\rho = \rho_0 + AT^2$. (b) Film thickness (t) dependence of the ρ_0 and A values.

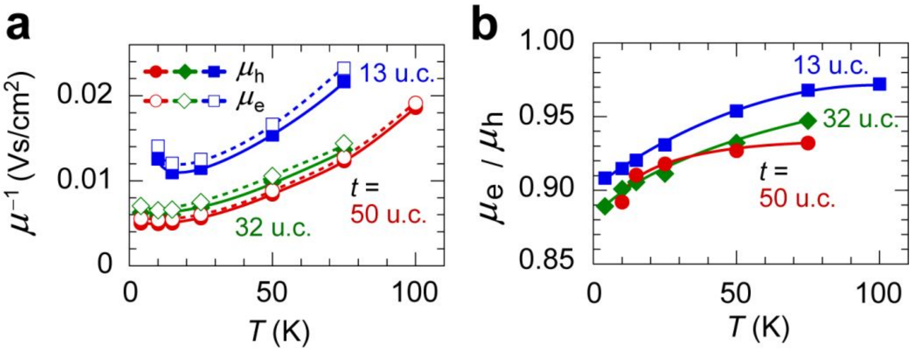

Figure S12. (a) Inverse of electron and hole mobilities (μ_e and μ_h) vs. temperature (T) with linear scale and (b) T dependence of μ_e and μ_h ratio, μ_e/μ_h for metallic LNO films with t = 13 u.c., 32 u.c., and 50 u.c., obtained from the result of the two carrier model analysis.

Thermal conductivity of $LaAlO_3$ and $LaNiO_3$

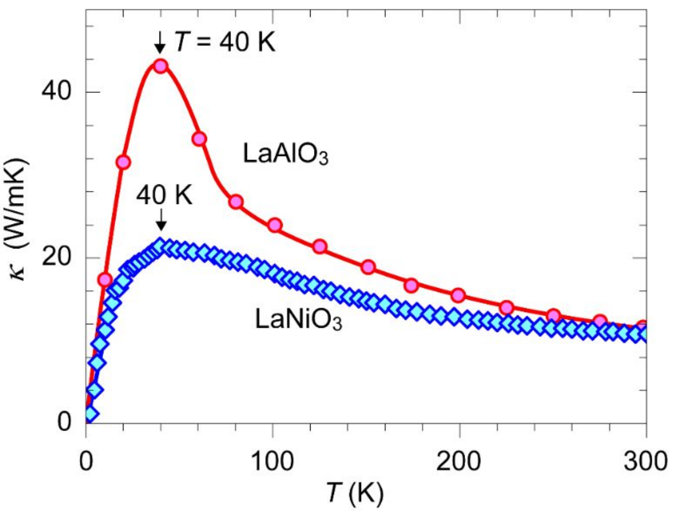

Figure S13. Temperature dependences of the thermal conductivity (κ) for bulk $LaAlO_3$ (red symbols)[22] and $LaNiO_3$ (blue symbols).[23]

REFERENCES

(1) Xu, X. Q.; Peng, J. L.; Li, Z. Y.; Ju, H. L.; Greene, R. L. Resisitivity, thermopower, and susceptibility of *R*NiO_3 (*R*=La,Pr). *Phys. Rev. B* **1993**, 48, 1112−1118.

(2) Gauquelin, N.; Benckiser, E.; Kinyanjui, M. K.; Wu, M.; Lu, Y.; Christiani, G.; Logvenov, G.; Habermeier, H.-U.; Kaiser, U.; Keimer, B.; Botton, G. A. Atomically resolved EELS mapping of the interfacial structure of epitaxially strained $LaNiO_3/LaAlO_3$ superlattices. *Phys. Rev. B* **2014**, 90, 195140.

(3) Tadano, T.; Gohda, Y.; Tsuneyuki, S. Anharmonic force constants extracted from first-principles molecular dynamics: applications to heat transfer simulations. *J. Phys.: Condens. Matte*r **2014**, 26, 225402.

(4) Giannozzi, P. et al., QUANTUM ESPRESSO: a modular and open-source software project for quantum simulations of materials. *J. Phys.: Condens. Matter* **2009**, 21, 395502.

(5) Perdew, J. P.; Burke, K.; Ernzerhof, M. Generalized gradient approximation made simple. *Phys. Rev. Lett.* **1996**, 77, 3865.

(6) Cao, Y.; Liu, X.; Kareev, M.; Choudhury, D.; Middey, S.; Meyers, D.; Kim, J.-W.; Ryan, P. J.; Freeland, J. W.; Chalhalian, J. Engineered Mott ground state in a $LaTiO_{3+\delta}/LaNiO_3$ heterostructure. *Nat. Commun.* **2016**, 7, 10418.

(7) Green, R. J.; Haverkort, M. W.; Sawatzky, G. A. Bond disproportionation and dynamical charge fluctuations in the perovskite rare-earth nickelates. *Phys. Rev. B* **2016**, 94, 195127.

(8) Golalikhani, M.; Lei, Q.; Chandrasena, R. U.; Kasaei, L.; Park, H.; Bai, J.; Orgiani, P.; Ciston, J.; Sterbinsky, G. E.; Arena, D. A.; Shafer, P.; Arenholz, E.; Davidson, B. A.; Millis, A. J.; Gray, A. X.; Xi, X. X. Nature of the metal-insulator transition in few-unit-cell-thick $LaNiO_3$ films. *Nat. Commun.* **2018**, 9, 2206.

(9) Gayathriy, N.; Raychaudhuri, A. K.; Xu, X. Q.; Peng, J. L.; Greene, R. L. Electronic conduction in $LaNiO_{3-\delta}$: the dependence on the oxygen stoichiometry δ. *J. Phys.: Condens. Matter* **1998**, 10, 1323−1338.

(10) Gauquelin, N.; Benckiser, E.; Kinyanjui, M. K.; Wu, M.; Lu, Y.; Christiani, G.; Logvenov, G.; Habermeier, H.-U.; Kaiser, U.; Keimer, B.; Botton, G. A. Atomically resolved EELS mapping of the interfacial structure of epitaxially strained $LaNiO_3/LaAlO_3$ superlattices. *Phys. Rev. B* **2014**, 90, 195140.

(11) Gray, A. X.; Janotti, A.; Son, J.; LeBeau, J. M.; Ueda, S.; Yamashita, Y.; Kobayashi, K.; Kaiser, A. M.; Sutarto, R.; Wadati, H.; Sawatzky, G. A.; Van de Walle, C. G.; Stemmer, S.;

Fadley, C. S. Insulating state of ultrathin epitaxial $LaNiO_3$ thin films detected by hard x-ray photoemission. *Phys. Rev. B* **2011**, 84, 075104.
(12) Snyder, G. J.; Toberer, E.S. Complex thermoelectric materials. *Nat. Mater.* **2008**, 7, 105−114.
(13) Ardizzone, I.; Zingl, M.; Teyssier, J.; Strand, H. U. R.; Peil, O.; Fowlie, J.; Georgescu, A. B.; Catalano, S.; Bachar, N.; Kuzmenko, A. B.; Gibert, M.; Triscone, J.-M.; Georges, A.; van der Marel, D. Optical properties of $LaNiO_3$ films tuned from compressive to tensile strain. *Phys. Rev. B* **2020**, 102, 155148.
(14) Behnia, K.; Jaccard, D.; Flouquet, J. On the thermoelectricity of correlated electrons in the zero-temperature limit. *J. Phys.: Condens. Matter.* **2004**, 16, 5187−5198.
(15) Hamada, N. Electronic band structure of $LaNiO_3$. *J. Phys. Chem. Solids* **1993**, 54, 1157−1160.
(16) Lee, S. B.; Chen, R.; Balents, L. Balents, Metal-insulator transition in a two-band model for the perovskite nickelates. *Phys. Rev. B* **2011**, 84, 165119.
(17) Eguchi, R.; Chainani, A.; Taguchi, M.; Matsunami, M.; Ishida, Y.; Horiba, K.; Senba, Y.; Ohashi, H.; Shin, S. Fermi surfaces, electron-hole asymmetry, and correlation kink in a three-dimensional Fermi liquid $LaNiO_3$. *Phys. Rev. B* **2009**, 79, 115122.
(18) Chasapis, T. C.; Koumoulis, D.; Leung, B.; Calta, N. P.; Lo, S.-H.; Dravid, V. P.; Bouchard, L.-S.; Kanatzidis, M. G. Two-band model interpretation of the p- to n-transition in ternary tetradymite topological insulators. *APL Mater.* **2015**, 3, 083601.
(19) Xu, X. Q.; Peng, J. L.; Li, Z. Y.; Ju, H. L.; Greene, R. L. Resisitivity, thermopower, and susceptibility of RNiO$_3$ (R=La,Pr). *Phys. Rev. B* **1993**, 48, 1112−1118.
(20) Son, J.; Moetakef, R.; LeBeau, J. M.; Ouellette, D.; Balents, L.; Allen, S. J.; Stemmer, S. Low-dimensional Mott material: Transport in ultrathin epitaxial $LaNiO_3$ films. *Appl. Phys. Lett.* **2010**, 96, 062114.
(21) Sreedhar, K.; Honig, J. M.; Darwin, M.; McElfresh, M.; Shand, P. M.; Xu, J.; Crooker, B. C.; Spalek, J. Electronic properties of the metallic perovskite $LaNiO_3$: Correlated behavior of 3d electrons. *Phys. Rev. B* **1992**, 46, 6382.
(22) Langenberg, E.; Ferreiro-Vila, E.; Leborán, V.; Fmega, A. O.; Pardo, V.; Rivadulla, F. Analysis of the temperature dependence of the thermal conductivity of insulating single crystal oxides. *APL Mater.* **2016**, 4, 104815.
(23) Zhou, J.-S.; Jin, C.-Q.; Long, Y.-W.; Yang, L.-X.; Goodenough, J. B. Anomalous electronic state in $CaCrO_3$ and $SrCrO_3$. *Phys. Rev. Lett.* **2006**, 96, 046408.
